# Supplementary figures and images for: Child and Maternal Factors Associated with Feeding Practices in Children with Poor Growth
Source: Nutrients. 2023 Nov 20;15(22):4850. doi: 10.3390/nu15224850 (PMC10675486; doi:10.3390/nu15224850)

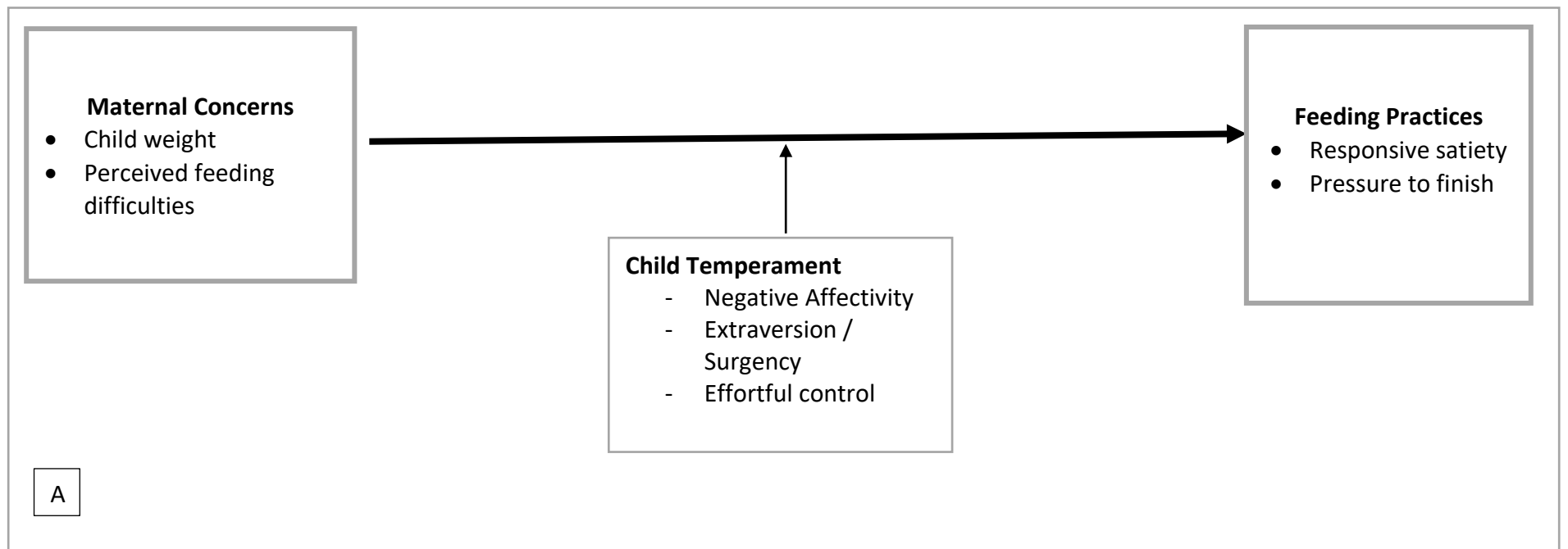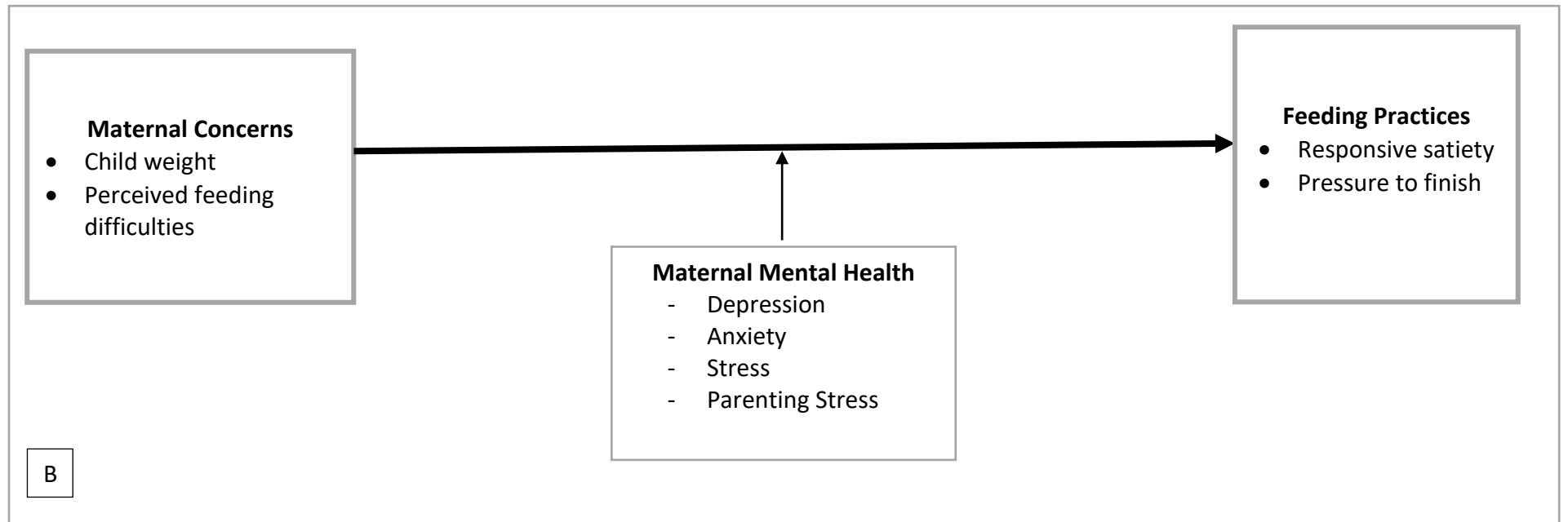

Supplement: Supplementary file 1 [file nutrients-15-04850-s001.zip › nutrients-2693875-supplementary.pdf]
